# Chimeric antigen receptor (CAR) immunotherapy: basic principles, current advances, and future prospects in neuro-oncology

**DOI:** 10.1007/s12026-021-09236-x

**Published:** 2021-09-23

**Authors:** Hyeon Joo Yoo, Biyan Nathanael Harapan

**Affiliations:** 1 Department of Internal Medicine V, Heidelberg University Hospital, 69120 Heidelberg, Germany; 2 Department of Neurosurgery, University Hospital, Ludwig-Maximilians-University of Munich, 81377 Munich, Germany

**Keywords:** Chimeric antigen receptor, CAR, T cells, Immunotherapy, Adoptive cell therapy, Neuro-oncology

## Abstract

With recent advances, chimeric antigen receptor (CAR) immunotherapy has become a promising modality for patients with refractory cancer diseases. The successful results of CAR T cell therapy in relapsed and refractory B-cell malignancies shifted the paradigm of cancer immunotherapy by awakening the scientific, clinical, and commercial interest in translating this technology for the treatment of solid cancers. This review elaborates on fundamental principles of CAR T cell therapy (development of CAR construct, challenges of CAR T cell therapy) and its application on solid tumors as well as CAR T cell therapy potential in the field of neuro-oncology. Glioblastoma (GBM) is identified as one of the most challenging solid tumors with a permissive immunological milieu and dismal prognosis. Standard multimodal treatment using maximal safe resection, radiochemotherapy, and maintenance chemotherapy extends the overall survival beyond a year. Recurrence is, however, inevitable. GBM holds several unique features including its vast intratumoral heterogeneity, immunosuppressive environment, and a partially permissive anatomic blood–brain barrier, which offers a unique opportunity to investigate new treatment approaches. Tremendous efforts have been made in recent years to investigate novel CAR targets and target combinations with standard modalities for solid tumors and GBM to improve treatment efficacy. In this review, we outline the history of CAR immunotherapy development, relevant CAR target antigens validated with CAR T cells as well as preclinical approaches in combination with adjunct approaches via checkpoint inhibition, bispecific antibodies, and second-line systemic therapies that enhance anticancer efficacy of the CAR-based cancer immunotherapy.

## The rise of immuno-oncology

In the 1980s, a new chapter of cellular immunotherapy was established for cancer patients, as the initial successful clinical applications of adoptive cell transfer in patients with metastatic melanoma and relapsed leukemia revealed the potential of a therapeutic approach with tumor-specific T cells [1–4]. Gene transfer techniques and the focus on selecting and expanding naturally occurring T cells found in patients or healthy donors were shared features of late twentieth century approaches [5]. Adoptive cell therapy (ACT) with tumor-infiltrating lymphocytes (TILs), T cell receptor (TCR)-modified T cells, and chimeric antigen receptor (CAR) T cells represent pioneer strategies. Nevertheless, TIL-based therapy showed major limitations as the process to harness TILs incited crucial challenges in logistical manner, whilst achieving limited results in selected highly immunogenic cancer entities (e.g. malignant melanoma) in terms of patient-specific treatment [6–8]. As an alternative to TIL-based therapy, T cell receptor (TCR) was genetically engineered to confer the specificity to a particular tumor target. Depending on the expression of human leukocyte antigen (HLA), T cells are generally restricted in their antigen recognition, which leads to limitations concerning application of TCR-modified T cells. Of note, tumors escape from immune surveillance of endogenous T-cell repertoire through downregulation of HLA-expression [9]. Genetic modification of autologous T cells by introduction of TCRs using either viral-mediated transduction of retrovirus [10–12], lentivirus [13–16], or nonviral gene transfer of DNA plasmids [17–31] or in vitro-transcribed mRNA species [32, 33], aptly named “chimeric antigen receptor” transgene, combines the functional dynamics of T cells with the antigen-specificity of an antibody to augment T cell function [5, 34, 35]. After infusion, CAR T cells may stimulate immune surveillance to prevent tumor recurrence through trans- as well as auto-costimulation and thus responding to tumor cells lacking costimulatory ligands [36, 37]. Since our immune system is programmed to avoid autoreactive immune responses [38], antitumor responses are frequently transient and ineffective as most tumor antigens are self-antigens that are also present in normal tissues [39], and host immune responses are evolved to prevent autoimmunity [40]. This aspect represents the main challenge in immuno-oncology, while CAR technology in the sense of T cell engineering provides a mean to overcome immune tolerance. The direct binding of CAR to antigen induces a competent activation signal, proliferation, and cytokine production independent of major histocompatibility complex (MHC) with extended applicability to multiple types of cancer [41–43].

## Development of CAR construct

**Fig. 1:**
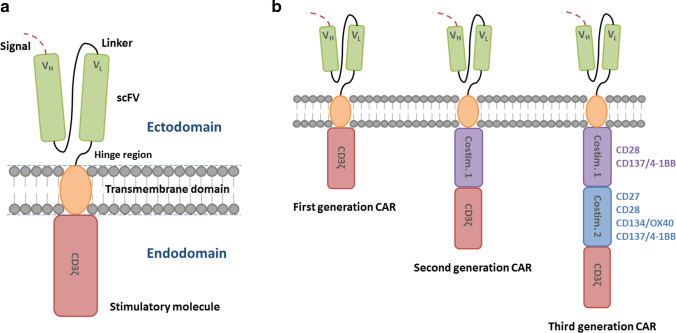
**A** Chimeric antigen receptor (CAR). CARs consist of four main components: the single-chain fragment variant (scFv), an extracellular spacer domain (hinge region), a transmembrane domain, and an intracellular signaling domain (stimulatory molecule: CD3ζ). V_H_, heavy chain variable region. V_L_, light chain variable region. **B** Evolution of chimeric antigen receptor (CAR). According to the evolution of CARs, the sophistication of the receptor has grown over time. They are referred to as the first, second, and third CARs, depending on the structure of their intracellular T cell region. Costim., costimulatory domain/element

Chimeric antigen receptors (CARs) are synthetic receptors which recognize and target cells expressing a cognate target ligand and as a result redirect the killing activity of CAR T cells against a specific tumor cell antigen [44]. CAR constructs consist of four main components: the single-chain variable fragment (scFv), the hinge, the transmembrane (TM) domain, and the intracellular signaling domain [45] (Fig. 1A). The CAR concept was originally reported by a Japanese group in 1987 [46] and further studied and developed at the Weizmann Institute by Zelig Eshhar, thereby establishing the first generation CARs [47, 48]. The potency of CAR signaling was subsequently improved by addition of costimulatory domains by Michel Sadelain, from whom the second, third, and fourth generation CARs were developed [35, 49, 50] (Fig. 1B). By identifying and evaluating the critical role of 4-1BB (CD 137) costimulation, Carl June’s group essentially contributed to expand the knowledge on second and third generation CARs [51–55].

Each of the four components has a distinct function and has been optimized through many variations of the constituent protein domains to maximize tumor detection, T cell activation, and tumor elimination [56].

The specificity of a CAR is conferred by its ectodomain derived from the antigen binding of a monoclonal antibody (mAb) with a heavy (*V*_*H*_) and light (*V*_*L*_) variable fragment connected by a flexible linker to construct a single-chain fragment variable (scFv) region for HLA-independent antigen recognition [46]. The order of variable fragments and the length of the linker have an impact on the antigen-binding affinity and the stability of the construct [57, 58]. The length and composition of the spacer domain determine the optimal distance between effector and target cell and are essential for immunological synapse formation [59, 60] and additional stability [61] by influencing CAR T cell function independent from the intracellular domain [62]. The spacer domain is commonly composed of amino acid sequences from CD28 or CD8α as well as CH2 and CD3 domains from IgG1, 2, or 4 [45, 57, 63].

The transmembrane domain consists of a hydrophobic alpha helix, which anchors the CAR construct. The TM region affects the degree of cell activation, hence, the functionality of CAR. CD28-derived TM domains are more prone to trigger activation-induced cell death (AICD) in T cells, whilst a CD3ζ-derived ones facilitate CAR dimerization with endogenous TCRs, thereby inducing T cell activation [64, 65].

The CAR design has been developed over generations. Its evolution has primarily focused on optimizing the intracellular signaling domains. The first-generation CARs only contained an activating domain, namely, CD3ζ, without a costimulatory domain. They showed limited cytokine production, insufficient T cell proliferation, and expansion, and rapidly became anergic [35, 45, 66, 67]. The clinical application of the CD3ζ-based CAR T cells in patients suffering from ovarian cancer [68], neuroblastoma [69], and non-Hodgkin’s lymphoma (NHL) [18] revealed limitations. The second and third generation CARs contain one or two costimulatory domains, respectively [70, 71], which enhance proliferation and exhibit antiapoptotic functions in human primary T cells [72] by directing the expansion of functional T cells on repeated exposure to antigen [49]. The second generation CARs achieved long-term persistence, expansion, and protection from AICD through integration of a costimulatory domain such as CD27 [73], CD28 [74, 75], CD134 (OX40) [76], or CD137 (CD4-1BB) [77, 78]. By combining the advantageous aspects of costimulatory domains, the third generation CARs emerged with greater intracellular signaling activity as well as superior persistence and proliferation properties [79, 80]. Kinetic and quantitative differences in CARs with different signaling domains have been demonstrated, highlighting that the choice of costimulatory signal has proven to be a critical element of CAR design [81].

## CAR T cell therapy

Adoptive transfer of autologous CD19-targeted CAR T cells was approved by the US Food and Drug Administration (FDA) as the first therapeutic approach with a genetic engineering component [82, 83] due to remarkable response rates, particularly in patients with diffuse large B cell lymphoma (DLBCL) or acute lymphoblastic leukemia (ALL) [84–90]. The first protocols either used gamma-retroviral or lentiviral vectors including either CD28- or 4-1BB-containing constructs [73, 82, 83, 91, 92]. Additional tumor antigen targets such as B cell maturation antigen (BCMA, also known as CD269) for treatment of multiple myeloma [93] have recently been discovered and are currently being evaluated. Further clinical approaches confirmed the data in larger series [5, 85, 86, 94].

In comparison to CD20 or CD22, CD19 is most commonly chosen as a target antigen [95, 96] due to its frequent, broader, and greater expression in B-cell leukemia and lymphoma in relation to other potential targets.

**Fig. 2:**
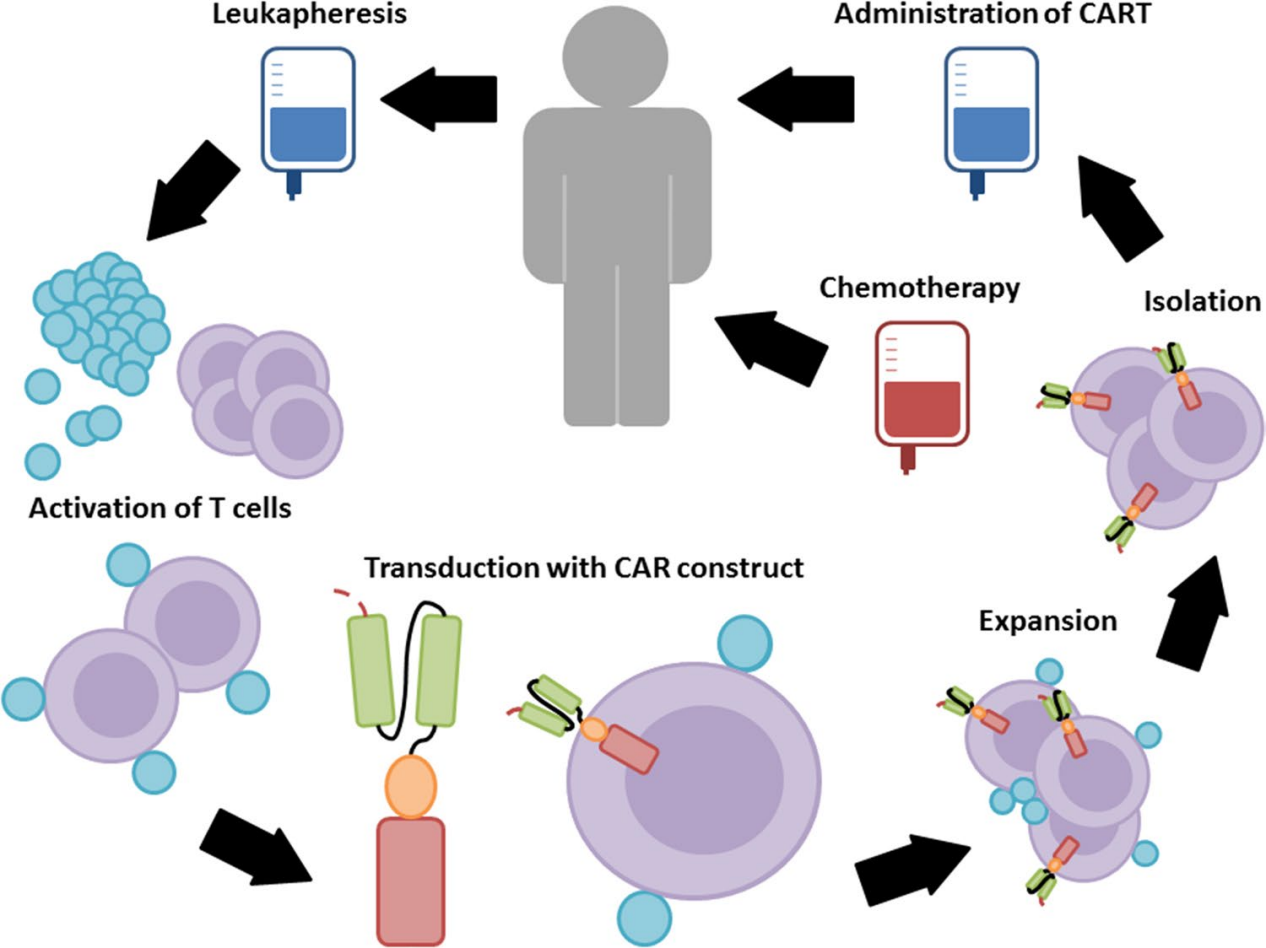
Manufacture of chimeric antigen receptor (CAR) T cells. Primarily, autologous T cells are isolated through leukapheresis and genetically modified ex vivo to express CARs, followed by the expansion in culture. After gene transfer of a CAR vector, the CAR T cells are expanded. Magnetic bead-based artificial antigen-presenting cells, which were used to activate T cells, are subsequently removed from the culture to isolate CAR T cells. The final CAR T cell culture is washed, concentrated, and subjected to end-of-process formulation with quality control testing and cryopreservation. Patients usually receive a lymphodepletion prior to the ultimate CAR T cell administration

The manufacturing time, financial burden, and severe toxicities associated with CAR T cell therapy represent current limitations [97]. Generation of an autologous CAR T cell product from chemorefractive patients is particularly limited in the quality of the obtained T cells as well as in the survival time of the patients (Fig. 2). The patients are required to be treatment-free for 2 weeks prior to apheresis to ensure sufficient cell numbers and qualitative viability for the manufacturing process, which takes around 2–4 weeks [88]. The alternative treatment with allogeneic CAR T cell products carries the critical risk of graft-versus-host-disease (GvHD), a life-threatening condition with rapid elimination of CAR T cells by the host immune system [98].

The spectrum of adverse effects associated with CAR T cells includes on-target effects, depending on the specificity of antibody scFv and T cell activation. These toxic effects are reversible once the target cells are eliminated, or the CAR T engraftment is terminated. In contrast, off-target toxic effects can be promoted by transduced T-cell population that undergo antigen-independent activation or unexpectedly attacks an antigen other than the intended one, possibly causing significant clinical long-term consequences, e.g., cardiac toxicity [99, 100]. This phenomenon is unrelated to toxicity due to other lines of treatment. To minimize the risk of off-target activation, the spacer domain of CARs can be modified, thereby avoiding an unintended initiation of an innate immune response [101].

Another potential side effect that poses a limitation of CAR technology is on-target/off-tumor toxicity, which occurs when CAR T cells attack nonpathogenic tissue that express the target antigen and therefore react with antigen different from the one intended. This is largely due to the fact that many targets of CAR T cells are also expressed on normal, non-tumor cells, leading to on-target/off-tumor toxicity to some extent through engagement of target antigen on nonpathogenic tissues [100, 102].

CAR T cell therapy can cause a clinical syndrome termed “cytokine release syndrome” (CRS) consisting of high fever, hypotension, hypoxia, tachycardia, and neurologic symptoms and complications associated with elevated levels of serum cytokines [85, 86, 103–105]. This syndrome seems to be related to both CD19 and BCMA CARs, while the severity correlates with tumor burden as measured by blasts in bone marrow at the time of therapy [106–108]. On-target CAR T cell activation leads to massive proliferation as well as release of high levels of cytokines and chemokines such as IL-6, IFN-γ, GM-CSF, and soluble IL-6R and IL-2Rα. Tocilizumab (Actemra), an IL-6-receptor antagonist, is an effective antibody recently approved by the FDA for the treatment of severe cytokine release syndrome caused by CAR T cells. It may lead to rapid resolution of severe CRS after blockade of IL-6R [90, 107]. As an alternative, glucocorticoid can be administered if patients do not respond well to IL-6R blockade [84, 85, 89, 107, 109, 110].

Neurotoxicity, immune effector cell-associated neurologic syndrome (ICANS), has been observed to develop after CRS and appears to be an adverse effect related to, among others, CD19-directed therapies [111, 112] since a similar spectrum of toxic effects have been observed with blinatumomab, which is a bispecific anti-CD19 and anti-CD3 monoclonal antibody [113]. The symptoms range from encephalopathy to expressive aphasia and seizures [86, 114, 115]. Even though the cause remains largely unknown, they are usually reversible and not associated with the spread of cancer to the central nervous system (CNS). Rare cases of cerebral edema have been reported in some trials [114, 115]. Until the pathophysiology of the neurologic syndrome is fully explained, the ultimate management remains primarily empirical and has mainly been directed towards management of CRS by suppression of T cell activation with corticosteroids and symptomatic and supportive care to maintain organ function [115].

Multi-antigen-targeted CAR T cells provide a strategy to expand the CAR T cell therapeutic window and overcome limitations by antigen escape and on-target/off-tumor toxicities by simultaneously targeting multiple surface antigens [116]. For instance, OR-gate CARs provide two scFv domains against various targets, which are either bound to a single TM and intracellular domain (“tandem CAR”) or represent two complete CAR constructs expressed on the same cell (“dual CAR”), aiming to prevent tumor escape [117–119]. AND-gate CARs also feature two scFvs, however, they require both antigens on the same cell prior to the ultimate signal propagation, from which tumor specificity is achieved by the dual expression of both antigens (“combinatorial CAR”) [120–122]. Other components of the immune system can be recruited and activated via additional expression of costimulatory ligands, such as 4-1BB-L [123] and CD40-L [124] or proinflammatory cytokines. The structure of On-Switch CARs relies on a small molecule, which assembles the fragmented CAR construct to allow a controlled CAR activation through the administration of a drug, thereby allowing titrable pharmacologic regulation [125]. Universal CARs describe another type of fragmented CAR design, in which the antigen-specific region can be exchanged to facilitate the targeting of different cancer types through the same TM and intracellular signaling construct [126–129].

In addition to second and third generation CARs, the fourth generation of CAR construct was developed to shape the tumor environment by the inducible release of immune modifiers [130, 131]. Sometimes, also referred to as T cells redirected for universal cytokine-mediated killing (“TRUCKs”), the fourth generation of CAR T cells is a novel design for targeting solid tumors and carry a transgenic “payload.” They are armed with immune stimulatory cytokines [131], which consequently improve CAR T cell expansion and persistence with increased resistance towards immunosuppressive tumor microenvironment (TME) [132]. As transgenic cytokine expression potentially triggers bystander T cells, antigen-negative cancer cells at the target site can be eliminated. The cytokines studied to date include IL-7, IL-12 [133, 134], IL-15 [135], IL-18 [136, 137], and IL-21 [138]. As IL-15 supports the development of T-memory stem cells (T_scm_) that promote superior in vivo function and persistence without influencing regulatory T (T_reg_) cells, it was observed to have a great potential to improve the function of CAR T cells [139–141]. Moreover, tumor-targeted IL-12 secreting T cells were shown to become resistant against inhibitory signals mediated by T_reg_ cells [134], resulting in maintenance of the optimal therapeutic level and accumulation of cytokines in target tissues, and thus, in destruction of both TAA (tumor-associated antigens)-expressing and TAA-negative tumor cells [142, 143]. To adapt this approach in terms of a protocol in practical manner, exact toxicity profile with controlled release of cytokines for safe application of this strategy must be guaranteed [144]. Inhibitory CARs turn an immunosuppressive signal from a tumor cell into an activating signal by fusing the extracellular inhibitory domain, for instance, PD-1, to activate intracellular CAR domain [145]. To optimize safety profile of CAR T cells, a suicide gene switch can be triggered in the event of adverse effects [146].

Moreover, an alternative strategy to mitigate the limitations of CAR T cell therapy has been developed, in which natural killer (NK) cells are utilized instead of T cells. As target recognition mechanisms of NK cells differ from that of cytotoxic T-lymphocytes (CTLs), NK cells, as innate lymphoid cells, receive activating and inhibitory signals by their germline-encoded receptor repertoire with the ability to recognize the absence of HLA-proteins. Thus, CAR T cells and CAR NK cells differ in various attributes. CAR T cells may be more effective in killing tumors and are more persistent in vivo, whereas CAR NK cells may offer a more favorable safety profile by combining natural anti-tumor function of NK cells with CAR-redirected function in terms of an intrinsic killing capacity of malignant cells with only a few side effects post-transplantation, such as limited CRS risks. However, they do not last as long and tend to require repeated administrations. Most CAR T cell-based approaches consist of autologous enriched T cells, whereas CAR NK cell-based gene therapy products can be generated from allogeneic donors, so that they possibly reduce the tremendous costs and the limited availability of an autologous therapy caused by logistics and the low cell numbers of heavily pretreated patients [147–149].

## Solid tumors

Modern attempts have recently been made to strive for successful treatment for solid tumors. Since these tumor entities rarely express specific target antigens, tumor-associated antigens that are enriched on most solid tumors are aimed as targets. The antigens studied so far include CEA, ERBB2, EGFR, GD2, CD33, CD123, mesothelin, MUC1, PSMA, PSCA, STn, and others [150–155]. However, since they are also expressed at low levels in normal tissues, the potential risk of significant on-target/off-tumor toxicity is increased [156–160].

The main challenges when targeting solid tumors are generally associated with the complicated structure and cellular milieu of solid tumors, which leads to inefficient penetration of CAR T cells into tumors [161, 162]. Even in the setting of a uniformly expressed TAA, there is still a great possibility of antigen loss or antigen escape when target antigen disappears from the surviving tumor [135, 159, 163–165], or the highly immunosuppressive nature of TME overcome CAR T cell activation and persistence [166].

Antigen heterogeneity poses a major obstacle to the use of CAR T cells for treatment of solid malignancies. The term refers to the different cells of the same tumor expressing different antigens. Thus, if only one antigen is targeted by CAR T cells, tumor cells negative for that specific antigen will escape [167].

CAR T cell-mediated lysis can further release tumor-specific neoantigens or epitopes that might be processed and presented by APCs to TILs, resulting in a secondary immune response that bolsters the efficacy of CAR T cells against tumor entities such as melanoma [168], NSCLC [169], malignant pleural mesotheliomas [170], pancreatic cancers [171], and others.

Several strategies and mechanisms have been under development to deal with and overcome the antigen heterogeneity of solid tumors, including the aforementioned fourth generation CARs with different construct designs. Anti-EGFR BiTEs were observed to increase the efficacy of antifolate receptor-α CAR T cells in preclinical models of the ovarian, colon, or pancreatic cancer and of anti-EGFRvIII CAR T cells in mouse models of GBM, providing the rationale to test this therapy in human trials with patients with solid tumors [172, 173]. Notably, universal CARs have been created, for which adapter elements are required as ligands to enable the targeting of multiple antigens with a single CAR T cell population. Different strategies play a significant role to successfully target heterogeneous solid tumors whilst minimizing possible off-tumor toxicities.

Numerous engineering strategies have already been implemented to improve and enhance CAR T cell trafficking in solid tumors. To avoid the challenges and adverse effects that arise from systemic application, CAR T cells have been injected directly intratumorally, among others in brain tumors [174], breast cancer [175], pleura mesothelioma [170], and liver metastases [176]. This approach has shown promising responses with the potential to limit on-target/off-tumor toxicities [177]. The regional application allows CAR T cells to expand and traffic to other tumor sites and promote responses of endogenous immune effect against tumors. An antitumor humoral response with multiple additional antigens has also been observed in patients who received infusions of T cells transduced with mRNA encoding an anti-mesothelin CAR [170]. Although it has not yet been studied extensively, this result suggests the potential application of regional delivery of CAR T cells to initiate systemic anticancer immune responses. Since many metastatic solid tumors are not susceptible to localized therapy, efforts to engineer CAR T cells with an intrinsic ability to optimize trafficking to sites of disease are of utmost importance.

Chemokines are crucial factors that mediate immune cell trafficking [178]. Expression of the macrophage colony-stimulating factor 1 receptor (CSF-1R) in CAR T cells would promote the cells to become responsive to CSF-1, which is enriched in many solid tumors. Similarly, CCR2b, which is the receptor for CCL2, seems overexpressed in multiple types of solid tumors, enhancing the infiltration of anti-GD2 CAR T cells into neuroblastoma xenograft tumors [179] as well as anti-mesothelin CAR T cell infiltration into mesothelioma xenografts by more than 12.5-fold with increased antitumor efficacy [180]. Expression of CC-chemokine receptor 4 (CCR4), which is commonly expressed on T helper (T_h_) cells and T_reg_ cells, is typically activated via CC-chemokine ligand 17 (CCL17) and CCL22 secreted by Reed-Sternberg cells of Hodgkin lymphoma, enhancing both CAR T cell migration to tumors and antitumor efficacy in a mouse xenograft model of Hodgkin lymphoma [181].

Inhibition of the PD-1 pathway causes an essential clinical benefit in patients with certain types of cancer [182]. PD-1 is an immune-checkpoint receptor expressed on activated T cells and can bind to PD-L1 expressed by tumor cells as well as other cell types to adopt an exhausted phenotype. CAR T cells engineered to secrete antagonistic anti-PD-1 scFvs showed a synergistic improvement in functionality and prolonged survival in immunocompetent syngeneic mouse models of PD-L1-positive hematological or solid cancers [183] as well as in xenograft [183, 184]. Increased anticancer efficacy of CAR T cell therapy via coadministration of antibodies inhibiting the PD-1 pathway in preclinical models [185] and in patients with ALL or DLBCL has been shown [186, 187]. Successful strategies regarding combination of CAR T cells with established immune-checkpoint inhibitors or other prodrugs have been demonstrated thus far. For instance, increased levels of reactive oxygen species (ROS) in TME are exploited by using ROS accelerator named as PipFcB, which is specifically activated in cancer cells to induce further generation and accumulation of ROS, due to which the tumor cells are rather primed to undergo lysis than other surrounding normal cells [188]. Besides anti-PD-1 scFv-secretion, CAR T cells are able to counter the actions of adenosine in the TME, which activates adenosine receptor A_2A_ to inhibit T cell function [189, 190]. Inhibitory signals in the TME also represent a challenging problem for CAR T cells which needs to be resolved. CAR T cells have been engineered to express switch cytokine receptors which convert inhibitory signals present in the TME into proinflammatory signals [172, 191, 192] or dominant-negative TGFβ-receptors that increase the ability to infiltrate, proliferate and enhance cytokine secretion, resist exhaustion and induce tumor eradication [193].

Amino acids, oxygen, and other nutrients in the TME influence the metabolism, function, and differentiation of T cells [194, 195]. Rapidly proliferating cancer cells and T effector cells have a strong demand for amino acids. Arginine, for instance, plays an important role for T cell function. Consequently, the competition between cancer and T effector cells over arginine in the TME might result in suppression of antitumor activity of T cells, possibly leading to T cell anergy. Therefore, supplementing T cells with arginine can improve the survival capacity and antitumor activity of these cells [196]. Engineering T cell metabolic pathways to express the antioxidant enzyme catalase enables T cells to better resist oxidative stress in vitro [197].

Since antigen escape remains a major concern in CAR T cell therapy, recruitment and activation of endogenous immune cells can be necessary to propagate and modulate an efficient antitumor immune response. For instance, TRUCKs can increase activity of CAR T cells that secrete stimulatory cytokines to trigger proliferation as well as to enhance survival and antitumor efficacy, while simultaneously altering the immune milieu of solid tumors [130].

## CAR T cell therapy in neuro-oncology

In recent years, several CARs for GBM have been developed and tested in clinical trials (Table 1). Some results seem to be quite promising. As the most common and malignant primary brain tumor in adults, GBM is responsible for 3–4% of all cancer-related deaths with an extremely poor prognosis [202]. The current standard therapy for GBM includes maximal surgical resection with consecutive radiotherapy and adjuvant chemotherapy with temozolomide (TMZ). However, despite recent progress in conventional therapeutic approaches with improved overall survival, recurrence is essentially inevitable, indicating that a more aggressive local therapy is required.

**Table 1:** Completed clinical trials of CAR T cell therapy in patients with GBM

Target antigen	Reference	Study phase	Dosage of CAR T cells	Response	Clinical trial
EGFRvIII	[165]	I	One intravenous dose 1.75 × 10^8^ – 5 × 10^8^ CAR T cells	MOS 8 months	NCT02209376
	[198]	I/II	Two intravenous doses 6.3 × 10^6^ to 2.6 × 10^10^ CAR T cells per infusion with an interval of 2 h	MOS 6.9 months MPFS 1.3 months	NCT01454596
IL13Rα2	[199]	I	Intravenous infusions of 10^8^ CAR T cells on days 1, 3, and 5 for duration of 2 weeks; repetition of treatment after 3 weeks	MS after relapse 11 months	NCT00730613
	[200]	I	Locoregional injections of 1 × 10^8^ CAR T cells and IL-2 twice per week for 2 weeks	MOS 19.7 months	NCT01082926
HER2	[201]	I	≥ 1 intravenous infusions of 1 × 10^6^/m^2^ – 1 × 10^8^/m^2^ CAR T cells	MOS 24.5 months MPFS 3.5 months	NCT01109095

*MS* median survival, *MOS* median overall survival, *MPFS* median progression-free survival

Various GBM antigens have been found as a potential target for CAR T cells, from which epidermal growth factor receptor variant III (EGFRvIII), human epidermal growth factor receptor 2 (HER2), and interleukin-13 receptor alpha 2 (IL-13Rα2) have been clinically verified as effective targets of CAR T cell therapy for GBM [203].

Since the route of administration is an essential determinant in therapeutic success, the question arises which mode of delivery for CAR T cells targeting GBM is the most optimal. Considering that extracranial metastasis is rare in primary brain tumors such as GBM [204], is systemic or locoregional delivery of CAR T cells more advantageous?

As the most common delivery approach for hematological and solid cancers, systemic delivery in form of intravenous (IV) administration exhibits systemic toxicities. Intraventricular (ICV) and/or intratumoral/intracavitary (ICT) administration are locoregional delivery strategies which require the implantation of a catheter delivery device/reservoir placed during surgery. CAR T cells are delivered into the cerebrospinal fluid via the ventricular system in ICV administration, while CAR T cells are directly administered into the tumor or resected tumor cavity in ICT delivery [177]. Locoregional routes of delivery do not merely lead to decreased risk of systemic toxicities; there is clear evidence that local delivery seems to outperform systemic delivery in terms of efficacy and benefit. For instance, outstanding clinical efficacy was reported in a patient with recurrent multifocal GBM with a regression of all intracranial and spinal tumors after administration of CAR T cells targeting IL-13Rα2 by means of locoregional delivery (ICT and ICV) [174]. This clinical response endured for 7.5 months after the start of CAR T cell therapy. Another clinical trial further shows the limit of systemic administration of CAR T cells targeting EGFRvIII since objective tumor regression could not be induced and delayed progression or prolonged survival in patients with recurrent GBM was not achieved [198]. In contrast, effective antitumor immune response and safety of HER2-specific CAR-modified virus-specific T cells in patients with progressive GBM with no serious adverse events have been observed, with intravenous administration as the route of delivery [201]. However, serious adverse effects following intravenous HER2-specific CAR T cell infusion resulting in pulmonary distress, cardiac arrests, and ultimately death have also been reported in the current literature [156]. This off-tumor toxicity can be attributed to first-pass clearance of HER2-specific CAR T cells in the lung with consecutive release of inflammatory cytokines, causing pulmonary edema and toxicity. A subsequent cytokine storm then leads to multiorgan failure. To prevent such serious adverse effects, a locoregional delivery strategy may be pursued.

Of note, other potential GBM-associated targets for CAR T cell therapy are currently examined, among others ephrin type A receptor 2 (EphA2) [205, 206], CD 70 [207], the cancer stem cell antigen CD133 [208, 209], chondroitin sulfate proteoglycan 4 (CSPG 4) [210, 211], B7-H3 [212, 213], and podoplanin (PDPN) [214].

A combinatorial approach to enhance antitumor efficacy has been implemented in a human neuroblastoma preclinical model. The combination of CAR T cells with bevacizumab, a recombinant human monoclonal antibody that blocks angiogenesis by inhibiting vascular endothelial growth factor A (VEGF), has shown encouraging results [215]. The theoretical background of the study is that tumor-driven neo-angiogenesis reinforces an immunosuppressive microenvironment and influences treatment responses. By administering an additional antiangiogenic drug, tumor vasculature is transiently reprogrammed and bevacizumab-mediated TME remodeling maximizes CAR T cell functions by increasing their tumor infiltration capacity [216].

Apart from the application of CAR T cell therapy for GBM and neuroblastoma, pediatric brain tumors (e.g., medulloblastoma, ependymoma, high-grade gliomas) seem to have the potential to be treated with CAR T cell therapy [217]. A recent study validates intrathecal delivery of CAR T cells targeting EphA2, HER2, and IL-13Rα2 as an effective treatment for primary, metastatic, and recurrent medulloblastoma and ependymoma in mouse models [218].

Noteworthily, antigens targeted in GBM to date are either easily downregulated or not particularly tumor-specific, which poses a major problem in the immunotherapy for GBM.

An innovative strategy to overcome tumor heterogeneity has been demonstrated in a recent research article describing the use of chlorotoxin-directed CAR T cells to achieve broader and effective GBM targeting. Although not an antibody-based CAR T cell therapy, this method seems to mediate antitumor activity against established GBM xenografts while simultaneously exhibiting negligible off-target effects. Systemic toxicity was not observed in systemic (intravenous) and regional delivery of chlorotoxin-CAR T cells into healthy and tumor-bearing mice. Results from this study suggest a potent anti-GBM activity of CAR T cells using chlorotoxin as the targeting domain and thereby the potential to reduce antigen escape [219].

Significant challenges in the development of novel therapies are due to certain characteristic features of GBM in contrast to other solid tumors, including biological factors such as the intracranial location, blood–brain barrier, heterogeneity of the tumor, and the unique, immunosuppressive TME [220]. The immunosuppressive TME may suppress the activity and proliferation of CAR T cells by releasing certain inhibitory molecules, soluble factors, and/or cytokines which create physical and metabolic blockades [221]. Intertumor and intratumor heterogeneities of molecular, genetic, and cellular signatures result in tumor diversity, making GBM more challenging to target with a single antigen. Immune escape and constant immune tolerance also represent limiting factors of CAR T cell therapy.

On the whole, further studies are warranted to evaluate CAR T cell therapy in patients with brain tumors with respect to its antitumor efficacy, safety profile, and potential combinatorial approaches while simultaneously considering the most important translational challenges.

## Conclusions

In this review, we illustrate the opportunities and challenges of immunotherapies in solid tumors with particular emphasis on GBM and discuss new avenues and novel treatment strategies based on CAR T cells and combination therapy. Tumor-specific redirection of the exquisite lytic capacity of CAR T cell-based therapy has become a promising new treatment modality. Recent advances in research techniques that utilize adoptive cell therapy approaches are expected to bring about crucial developments regarding treatment options for patients suffering from hematologic malignancies and solid tumors, including brain tumors.

GBM thereby shows a particular challenge due to its aggressive nature and highly immunosuppressive TME. CAR T cell therapy has introduced and supported a crucial clinical development for treatment of malignant glioma.

The potential combination of CAR T cells with the investigated antigens, immune checkpoint inhibitors, or inhibitors of angiogenesis present effective strategies to additionally enhance the cytotoxic potential of CAR-engineered immunotherapy against solid tumors and improve their ability to modulate innate and adaptive immune cells in the complex TME. The preferred way to design receptor-targeted therapeutic approaches in neuro-oncology should, among others, include the combined targeting of multiple receptors.

Novel results highlight the potential advantage of locoregional/intraventricular application of CAR T cells compared to systemic application.

Further preclinical and clinical trials are needed to optimize CAR T cell treatment approaches and to harness the antitumor efficacy of CAR T cells combined with adjunct therapies (Table 2).

**Table 2:** Ongoing clinical trials of CAR T cell therapy in patients with GBM

Target antigen	Study phase	Dosage of CAR T cells	Enrolment/primary completion date	Sponsor	Clinical trial
EGFRvIII	I	CART-EGFRvIII + pembrolizumab	7/December 2020	University of Pennsylvania	NCT03726515
	I	Initial dose of 2.5 × 10^8^ CAR T cells per intracerebral infusion; dose escalation in successive cohorts	24/December 2021	Duke University	NCT03283631
IL13Rα2	I	IL13Rα2-specific, hinge optimized, 41BB/truncated CD19-expressing CAR T cells by locoregional, intracavitary, or intraventricular catheter; weekly for 3 weeks and additional infusion if eligible	92/January 2021	City of Hope Medical Center	NCT02206362
	I	Intravenous infusion of nivolumab and ipilimumab followed by intraventricular or locoregional infusion of CAR T cells up to four cycles	60/December 2022	City of Hope Medical Center	NCT04003649
B7-H3	I	Three locoregional or intracerebroventricular injections of CAR T cells at two doses between temozolomide cycles	12/May 2022	Second Affiliated Hospital, School of Medicine, Zhejiang University	NCT04385173
	I/II	Three locoregional or intracerebroventricular injections of CAR T cells at two doses between temozolomide cycles	40/June 2024	Second Affiliated Hospital of Zhejiang	NCT04077866
GD2	I	Intravenous injections of 1 × 10^7^ – 1 × 10^8^ CAR T cells with or without lymphodepletion chemotherapy	34/February 2023	Baylor College of Medicine	NCT04099797
MMP2	I	Three weekly cycles of one or two CAR T cell infusions	36/February 2023	City of Hope Medical Center	NCT04214392
CD147	I	Intracavity injection of CAR T cells once per week for 3 weeks	31/October 2020	Xijiang Hospital	NCT04045647
Variable	I	CAR T cells expressing receptors specific for EGFRvIII, IL13Rα2, Her2, CD133, EphA2, or GD2 with or without anti-PDL-1 mAb	100/January 2021	Xuanwu Hospital	NCT03423992

## Data Availability

Authors can confirm that all relevant data are included in the article. Dataset(s) derived from public resources and made available with the article (references).
